# Recent Application Prospects of Chitosan Based Composites for the Metal Contaminated Wastewater Treatment

**DOI:** 10.3390/polym15061453

**Published:** 2023-03-14

**Authors:** Ashoka Gamage, Nepali Jayasinghe, Punniamoorthy Thiviya, M. L. Dilini Wasana, Othmane Merah, Terrence Madhujith, Janardhan Reddy Koduru

**Affiliations:** 1 Chemical and Process Engineering, Faculty of Engineering, University of Peradeniya, Peradeniya 20400, Sri Lanka; 2 China Sri Lanka Joint Research and Demonstration Center for Water Technology, Peradeniya 20400, Sri Lanka; 3 Postgraduate Institute of Agriculture, University of Peradeniya, Peradeniya 20400, Sri Lanka; 4 Department of Agricultural Technology, Faculty of Technology, University of Colombo, Colombo 10200, Sri Lanka; 5 Laboratoire de Chimie Agro-Industrielle, LCA, Université de Toulouse, CEDEX 4, 31030 Toulouse, France; 6 Département Génie Biologique, IUT Paul Sabatier, Université Paul Sabatier, 32000 Auch, France; 7 Department of Food Science and Technology, Faculty of Agriculture, University of Peradeniya, Peradeniya 20400, Sri Lanka; 8 Department of Environmental Engineering, Kwangwoon University, Seoul 01897, Republic of Korea

**Keywords:** adsorption, chelation, chitosan, deacetylation, heavy metals, ion exchange

## Abstract

Heavy metals, known for their toxic nature and ability to accumulate and magnify in the food chain, are a major environmental concern. The use of environmentally friendly adsorbents, such as chitosan (CS)—a biodegradable cationic polysaccharide, has gained attention for removing heavy metals from water. This review discusses the physicochemical properties of CS and its composites and nanocomposites and their potential application in wastewater treatment.

## 1. Introduction

Various materials, including natural contaminants, fluoride, chloride, nitrate, iron, calcium, magnesium, and other contaminants from byproducts of agriculture and industries such as heavy metals, organic dyes, insecticides and fertilizers, spilled oils, batteries, diesel fuel, household chemicals such as synthetic detergents, and some pathogenic microbes can contaminate the ocean, groundwater and surface water bodies (rivers, lakes, ponds, reservoirs) [1,2,3,4,5,6]. Contaminated water will not be suitable for drinking, habitat, irrigation, recreation, and other industrial activities [4,7].

Heavy metal has gained significant importance in ecotoxicology due to their long persistence, bioaccumulation, and bio-magnification in the food chain [8]. Heavy metal ions are commonly discharged by various industrial activities, including electroplating, battery manufacturing, pesticide production, mining, nuclear power, textile manufacturing, and other similar industries [9,10,11]. The global average content of Cr, Mn, Co, Ni, As and Cd on surface water bodies exceeded the permitted level suggested by WHO and USEPA Guidelines [8]. Heavy metal ions, such as As, Cr, Ni, Co, Hg, Pb, U, and Cd, have become a major threat as they are toxic in nature and accumulate in food chains due to their non-biodegradability and cause various health-related issues even at trace levels. Therefore, in order to safeguard human health and the environment, it is crucial to eliminate heavy metals prior to their release into the surroundings [10,12,13,14,15].

Heavy metal removal can be accomplished through various methods, such as membrane separation, solvent extraction, chemical oxidation-reduction, chemical precipitate, reverse osmosis, flocculation, electrocoagulation, supercritical isothermal treatment, and adsorption [13,16,17]. Among these methods, adsorption has gained wide acceptance due to its high efficiency, simple handling, different adsorbent availability, and cost-effectiveness. In recent years, attention has shifted towards the use of low-cost adsorbents made from renewable resources, considering environmental and cost factors. CS, being a low-cost adsorbent derived from natural sources, has gained interest as a sorbent for heavy metal removal through adsorption in aqueous solutions [13,18]. Adsorbents for heavy metal removal have been widely fabricated from polysaccharides, including chitin/chitosan, cellulose, hemicelluloses, lignin, alginates, carrageenan, granular activated carbon, clays, silica, agricultural waste biomass, metal oxides, various Metal-Organic Frameworks (MOFs), and microbial biomass (probiotics and yeasts) [15,17,19,20,21,22,23].

**Figure 1:**
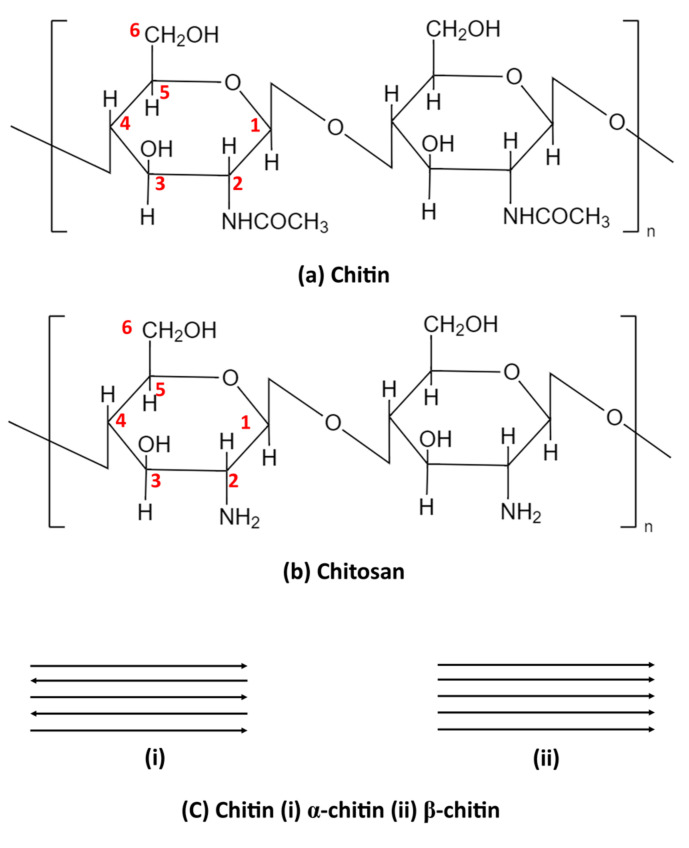
Chemical structure of (**a**) chitin and (**b**) CS (**c**) α- and β-chitin.

Chitin can be obtained from shellfish, including shrimp and crabs, and is recognized as the second most abundant polymer in nature, following cellulose [13,24]. Chitin, a linear polymer of N-acetyl-D-glucosamine, is one of the most abundant organic materials produced annually through biosynthesis in animals, especially in crustaceans, mollusks, and insects, as well as some fungi [25,26]. CS (poly[β-(1-4)-2-amino-2-deoxy-d-glucopyranose]) is a derivative of chitin, a polymer of D-glucosamine (Figure 1), which has primary reactive amino groups and hydroxyl groups and can chelate many metal ions. CS is mainly insoluble in water or organic solvents, but at acidic pH, CS becomes a water-soluble cationic polymer as amine gets protonated [25]. Chitin molecule mainly exists in two polymorphic structures: α-and β-chitin. Chitin from crustaceans, including shrimp, prawns, and crab shells, and fungi cell wall consists of an α-crystallographic structure in which the main chains are arranged in an antiparallel fashion with strong intermolecular hydrogen bonding. Chitin is found in diatoms, mollusks and squid pens and has a β-crystallographic structure, in which the chitin chains are arranged in a parallel way with relatively weak intermolecular forces [27,28,29,30]. CS is a natural polyamine made by deacetylating chitin, consisting of poly(β-(1,4)-2-deoxy-2-amino-d-glucose). The physicochemical properties of chitin and CS are affected by several factors, including the degree of acetylation, molecular weight, crystallinity degree, source, and processing method [31].

Today natural materials have emerged as cheaper, sustainable, and more efficient adsorbents in replacement for non-biodegradable synthetic material to treat contaminated water. In this aspect, CS is widely studied as they exhibit a range of characteristics, including biodegradability, non-toxicity, biocompatibility, abundance in nature, low cost, hydrophilicity, high content of functional groups (–NH_2_, –OH) that can contribute to various chemical modifications, attractive adsorption capacity, and metal ion chelation potential [22,32]. However, drawbacks of CS, including poor mechanical properties, pH sensitivity, low thermal stability, variability of polymer characteristics, poor solubility, low surface area, non-porosity, low adsorption capacity, and lack of reusability, limit their application in wastewater treatment [22,32,33,34]. The physical modification (transform powder form into nanoparticle) and chemical modification (crosslinking, graft modification, etc.) can improve the solubility and physical properties [32,35].

Chitosan has an excellent adsorption capacity for metal ions due to the presence of hydroxyl and amino functional groups, which can form complexes with metal ions via chelation, hydrogen bonding or electrostatic attraction [20]. The adsorption capacity of CS is found to be much higher than that of chitin because of its relatively higher amino groups. Additionally, CS-based composites have been extensively researched for their use in wastewater treatment [36,37,38]. In this context, this review focuses on the application of CS in wastewater treatment, mainly concerning extraction, physicochemical properties of CS, and application of CS on wastewater treatment.

## 2. Chitin Sources and Composition

To address environmental concerns, the industrial production of chitin and chitosan (CS) must be carried out on a large scale and at a competitive cost [39]. Chitin can be found in various sources, including crustacean exoskeletons (lobsters, shrimps, prawns, crabs, krill, crayfish), mollusks (octopus, cuttlefish, clams, oysters, squids, snails), algae (diatoms, brown algae, green algae), insects cuticles, and fungal cell walls [40]. Presently, crustacean waste, such as shrimp, crabs, prawns, and lobsters, is the primary source of industrial chitin [40,41]. Though, chitin/chitosan from crustaceans has limitations, such as limited raw material supply, a seasonal, higher concentration of CaCO_3_, requirement of chemical treatment, the possibility of heavy metal contamination, and consume long time. In contrast, chitin/chitosan fungal sources have seasonal independence, superior particle size, uniformity, lower molecular weight, and are free from heavy metals [42]. Table 1 outlines the proximate composition of several chitin sources, with shells comprising chitin, protein, minerals, and pigments [43]. Crustacean (crab, shrimp, and lobster) shell wastes consist of 15–40% chitin, 20–50% CaCO_3_, and 20–40% protein, along with lipids, pigments, and other minerals in small amounts [44]. Therefore, removing proteins, minerals, and pigments is necessary for chitin production [41].

## 3. Production of Chitosan from Chitin

**Figure 2:**
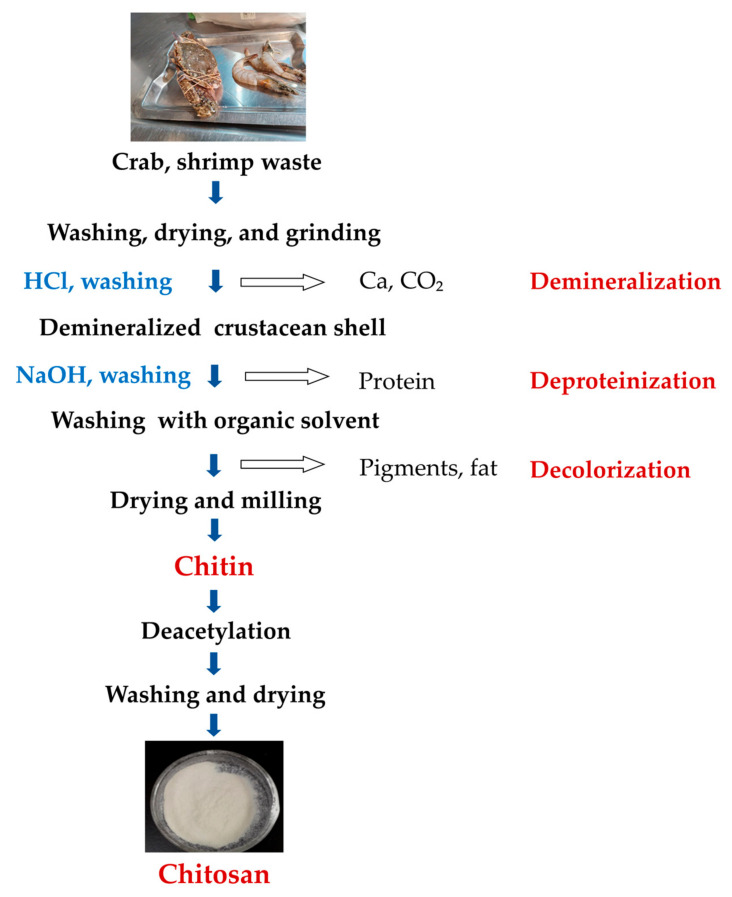
A simplified flow diagram of chitin and CS preparation [47,48].

The chitin production process can be achieved via four main steps: sample preparation, demineralization (removal of the mineral fraction, mainly CaCO_3_), deproteinization (removal of protein fraction), and discoloration (removal of carotenoid pigments) (Figure 2). In order to obtain pure chitin without impurities, demineralization, deproteinization, and decolorization are necessary. Demineralization is achieved using acid and deproteinization using alkali, while decolorizing agents such as chloroform, hydrogen peroxide, and acetone are used to remove pigments. It is important to correctly manipulate time, chemical concentration, and temperature to achieve the highest molecular weight of chitin/CS [46]. The deproteinization and demineralization steps can be carried out in reverse order [47], and CS can be produced by deacetylating chitin. A flow diagram for the isolation of chitin and the production of CS is shown in Figure 2.

### 3.1. Production of Chitin

#### 3.1.1. Preparation of Raw Materials

To remove any residual crustacean flesh, lipids, or other impurities, the shells were initially rinsed with hot tap water or boiling water with stirring using a mechanical stirrer [49]. After that, the shells were washed with hot distilled water and dried in an oven at 60 °C. The dried shells were either ground into small pieces or pulverized and sieved through meshes of size 60–120 µm. Grinding is a crucial step in order to increase the surface area and achieve uniformly-sized particles for the purpose of removing soluble organics and adhered proteins [46,49,50].

#### 3.1.2. Deproteinization

The process of deproteinization is challenging as it involves the breakdown of the chemical bonds that exist between chitin and proteins [51]. The removal of proteins from the grounded crustacean shell waste is achieved through treatment with a hot, weak NaOH solution (1–10%) at a temperature range of 65–100 °C. The deproteinization stage is challenging as it involves breaking down the chemical bonds between chitin and proteins. To achieve deproteinization, one can vary the alkali concentration, time, temperature, and solid-to-solvent ratios [48]. Deproteinization can be accomplished using various reagents that include NaOH, Na_2_CO_3_, NaHCO_3_, KOH, K_2_CO_3_, Ca(OH)_2_, Na_2_SO_3_, NaHSO_3_, CaHSO_3_, Na_3_PO_4_, and Na_2_S. Nonetheless, NaOH is the most commonly employed reagent for this process [48,52]. The use of chemical treatment can lead to a decrease in the molecular weight of chitin as it may cause partial deacetylation and depolymerization of the biopolymer through hydrolysis [51]. Therefore, chemical treatment is replaced by biological methods, including proteolytic enzymes and organic acids (lactic acid and acetic acid) produced by microbial fermentation. But, considering the possible microbial contamination and difficulty in achieving complete protein removal, chemical treatment is widely used in the deproteinization process [53].

#### 3.1.3. Demineralization

A high content of minerals, i.e., crustaceans’ exoskeleton contains more than 50% of CaCO3, requires a demineralization process [27]. The demineralization process involves the conversion of minerals, mainly CaCO_3,_ into soluble salts using acids (Equation (1)). Dilute HCl is the commonly used acid for demineralization, although other acids such as HNO_3_, H_2_SO_4_, CH_3_COOH, and HCOOH are also used in this process [48,51].

2 HCl + CaCO_3_ → CaCl_2_ + H_2_O + CO_2_ ↑ (1)

Following the production of soluble salts, the chitin solid phase can be filtered and subsequently washed using deionized water, enabling easy separation of the salts [51]. Antifoam can be used to control undesirable foam production due to CO_2_ generation [54].

The use of mild acids in the demineralization process can help minimize the degradation of chitin, as compared to the use of HCl, which may cause depolymerization and deacetylation of the native chitin [48]. The demineralization process varies based on factors such as the degree of mineralization, extraction time, temperature, particle size, acid concentration, and solute/solvent ratio. The stoichiometric amount of minerals requires two molecules of HCl to convert one molecule of CaCO_3_ into soluble salts (CaCl_2_), according to the equation. Due to heterogeneity, the complete removal of minerals is challenging and may require larger amounts or higher concentrations of acids [51]. Demineralization can be carried out by biological method, using acid (e.g., lactic acid) producing bacteria or enzymes, such as Alcalase^®^ [27].

#### 3.1.4. Discoloration

During the processes of deproteinization and demineralization, the resulting chitin product can take on a colored appearance due to the presence of carotenoids found in the exoskeleton of crustaceans [54]. Astaxanthin, astatine, canthaxanthin, lutein, and β-carotene are the main carotenoid components. To produce chitin that is white in color and has added commercial value, the process of discoloration is important. Two steps are involved in the discoloration of shellfish processing discards. The first step involves the use of necessary reagents to extract pigments, while the second step involves the use of appropriate chemicals for bleaching. Organic solvents such as acetone, chloroform, ether, or ethanol are typically employed for pigment extraction. Commonly used bleaching/whitening agents include sodium hypochlorite, potassium permanganate, and hydrogen peroxide [40,48]. After each step (deproteinization, demineralization, and discoloration) sample is collected by filtration, washing to neutralize, and drying [55].

### 3.2. Production of Chitosan from Chitin (Deacetylation)

Both enzymatic and chemical methods can be employed to convert chitin to CS. However, due to their low cost and suitability for mass production, chemical methods are commonly utilized for the commercial production of CS [51]. Converting chitin to CS can be achieved by partially or completely removing acetyl groups through treatment with concentrated alkali, such as NaOH or KOH (40–50%) at high temperatures (>100 °C) [48]. The use of acids in the deacetylation process is uncommon, as glycosidic bonds are highly susceptible to acid [51]. Finally, the CS can be separated by filtration, washing with distilled water to neutralize, and oven drying [46].

The deacetylation process has an impact on the molecular weight, degree of acetylation, and distribution of acetyl groups in the polymer chain. Several factors that influence the characteristics of CS include the type of alkali reagent used (NaOH being more efficient than KOH), its concentration, temperature, reaction time, repetition of deacetylation steps, atmospheric conditions (nitrogen or air), particle size, source of raw material, chitin/solvent ratio, and the use of a reducing agent (such as sodium borohydride) [51]. Table 2 summarize the degree of deacetylation (or acetylation) at different various process parameters. 

## 4. Characteristics of Chitosan

### 4.1. Molecular Weight

The N-deacetylation reaction results in molecular weight reduction [60]. Natural chitin typically has a molecular weight exceeding 1000 kDa. Commercially available CS usually ranges from 100–1000 kDa, and its molecular weight is affected by the preparation method and the source of raw materials [61,62]. For example, the molecular weight of CS from insects has reported lower values, ranging from 26–300 kDa [63]. Degradation of CS can be caused by processing factors such as elevated temperature, dissolved oxygen, and shear stress. High temperature or concentrated acids (e.g., HCl, CH_3_COOH) cause molecular weight changes, while EDTA results in minimal degradation [54]. Techniques such as HPLC and light scattering methods can be used to determine the molecular weight distributions of CS [60].

### 4.2. Degree of Deacetylation

Chitin is deacetylated by removing the acetyl group, resulting in amino groups (–NH_2_) that are highly chemically reactive [54]. The degree of deacetylation is a crucial determinant of the physicochemical properties and biodegradation of the material. When the degree of deacetylation of chitin is 50% or higher, it is generally known as CS [64]. The degree of deacetylation is the proportion of glucosamine monomers in the chitosan chain and can affect the solubility and performance of chitosan in various applications [63]. The degree of deacetylation can be determined using different tools, such as infrared (IR) spectroscopy or nuclear magnetic resonance (NMR) spectroscopy [46,60,63].

### 4.3. Solubility

The strong intra- and intermolecular hydrogen bonding and highly crystalline structure of chitin make it insoluble in water, dilute aqueous salt solutions, and most organic solvents [60,65]. In contrast, CS is a cationic polysaccharide soluble in dilute aqueous acidic solution (below pH 6) [64,65]. Protonation plays an important role in the solubility of CS in acids, such as CH_3_COOH and HCl, and the pH and the pK of the acid determine the degree of ionization on -NH_2_ functional group at C-2 of the d-glucosamine repeat unit [66].

Along with the pH, the ionic strength of the solvent determines the degree of protonation or charge density of the amines group, thereby, the solubility of CS. CS is soluble in acidic pH (<pH 6) and insoluble in neutral and basic pH [47]. Organic acids such as acetic, formic, and lactic acids are used for dissolving CS [54]. Table 3 summarizes the frequently used solvents for chitin and CS.

The solubility and applicability of CS depend on two primary factors: the degree of deacetylation and molecular weight. When the degree of acetylation is below 50%, CS obtained from partially deacetylated chitin can dissolve in acidic conditions [46].

The low deacetylated degree of CS (deacetylation degree of 55–70%) is almost completely insoluble in water. Whereas CS with the middle deacetylation degree (70–85%) is partly dissolved in water, and the high deacetylation degree (85–95%) of CS has good solubility in water. Moreover, the ultrahigh deacetylation degree of CS (95–100%) is difficult to obtain [69].

Because of the insolubility of CS in water (at neutral pH), it produces toxic compounds that can pose a threat to the environment. Therefore, CS can be modified to improve the solubility by attaching polar functional groups (-OH and -COOH) to the CS chain and modifying the molecular weight (solubility increased with decreased molecular weight) [70].

## 5. Neutralization and Chemical and Physical Modification of Chitosan

**Figure 3:**
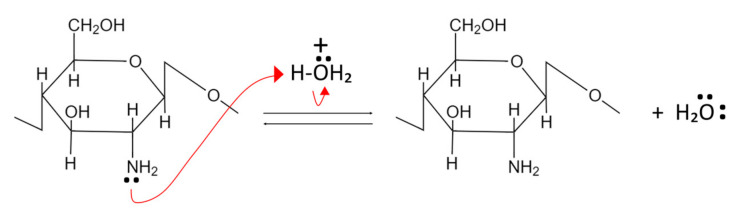
Neutralization reaction of chitin and CS.

Chitin and CS act as weak bases and undergo alkaline compound neutralization reactions. In this process, the primary amine group’s nonbonding pair of electrons from the glucosamine units accept protons and become positively charged, as shown in Figure 3 [67,68].

CS is not soluble at neutral and basic pH but can dissolve in acidic pH due to increased polarity and polymer-polymer electrostatic repulsion. In contrast, chitin remains insoluble even in acidic pH because it has fewer amino groups than CS [67].

Due to the presence of a non-bonding electron pair in the primary amino groups, CS functions as a potent nucleophile. Additionally, the primary amine groups at CS exhibit greater nucleophilic characteristics than the primary hydroxyl groups found at the C-6 position [67,68].

Due to the presence of the larger amount of amino (at C-2) and hydroxyl groups (at C-3 and C-6), CS can undergo various chemical modifications, such as acylation, carboxylation and etherification, hydroxylation, phosphorylation, thiolation, methylation, sulphonation, quaternization, crosslinking, graft copolymerization, chelation, oxidation, alkylation etc. This chemical modification generates various CS derivatives with improved solubility (in water or organic solvents), as well as other physicochemical and mechanical properties, which make them a potential material in many applications in various fields [65,68,71].

**Figure 4:**
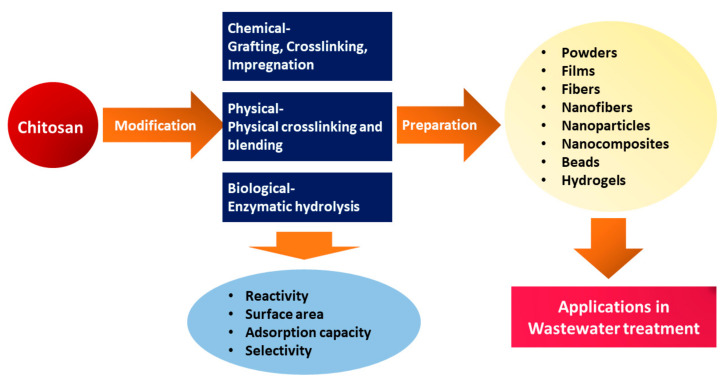
Schematic representation of modification of CS and CS-based materials preparation for wastewater treatment.

Furthermore, CS can be modified through various physical modifications into different forms, including solution, powder, flake, fiber, nanofiber, beads, and film, allowing for a wide range of applications across multiple fields, including water treatment (Figure 4) [72,73]. Moreover, to improve adsorption performance, CS has been modified into various forms, such as hydrogel, nanoparticles, and nanofibers, due to low specific area and porosity in powder and flake forms [74].

## 6. Waste Treatment and Purification of Water

### 6.1. Chitosan Based Composites to Eliminate Heavy Metal Ions

The non-biodegradability, carcinogenicity, and toxicity at low concentrations make organic dyes and heavy metals the most dangerous water pollutants [75]. Heavy metals, in particular, pose a significant risk to both human health and the environment as they are toxic and can accumulate and magnify in the food chain [12]. To ensure safe water consumption and human activities, it is crucial to eliminate heavy metals from the water before discharging them into the environment [12,75].

To remediate water, different techniques have been employed, such as flotation, coagulation/flocculation, oxidation-reduction, precipitation, membrane techniques (nanofiltration, ultrafiltration, reverse osmosis), ion exchange, electrochemical, photocatalytic degradation, and adsorption [61,76]. Among these methods, adsorption has a high removal rate, easy accessibility, low cost, and reduced secondary pollution [61,77]. Various parameters, such as pH, temperature, contact time, adsorbent dosage, initial metal concentration, and type of sorbent (surface area, porosity, functional group distribution), influence the metal adsorption process [78,79].

In recent years, there has been a growing interest in using CS-based adsorbents for removing heavy metals from water due to their excellent adsorption capacity, reusability, thermal stability, biodegradability, non-toxicity, low cost, and renewability [12].

CS can be used as a chelating agent as well as a flocculant [80]. Due to inter- and intra-molecular hydrogen bonding, CS is poorly soluble in neutral pH. It also has low antioxidant activity due to the absence of H atom donors and is characterized by high hydrophilicity, rigidity, and brittleness. However, these properties can be enhanced through chemical, mechanical/physical, or enzymatic modifications of CS [12].

Several cross-linking agents, including epichlorohydrin, 1,1,3,3-tetra methoxy propane, glycerolpolygglycidyl ether, chloromethyl oxirane, glutaraldehyde, ethylene glycol, diglycidyl ether and tri-polyphosphate, have been utilized [40,81]. The incorporation of new functional groups such as histidine, heparin, succinic anhydride, and N, O carboxymethyl into CS derivatives can improve their metal ion sorption selectivity and adsorption ability [81]. Modified shrimp-based CS, a natural scavenger of heavy metals, exhibited the maximum adsorption capacity of 20.4, 15.9, 7.0, and 6.3 mg/g for Cr, As, Ni, and Co, respectively [56].

Xanthate chitosan cross-linked with epichlorohydrin, which was chemically modified, achieved a maximum adsorption capacity of 43.47 mg/g at 50 °C, as reported in [80]. A CS-based flocculant, CMCTS-g-P(AM-CA), composed of carboxymethyl-CS, acrylamide, and ammonium di-thiocarbamate, was developed for removing heavy metals from wastewater. It exhibited high removal rates of 95.24% and 95.72% for Pb(II) and Cd(II), respectively [82]. Adsorbent fabricated from magnetic thiolated/quaternized-CS composite exhibited high removal efficiency (13.6–235.6 mg/g) for heavy metal ions, including As(V), As(III), Cu(II), Hg(II), Zn(II), Cd(II), and Pb(II), under neutral conditions [77].

**Figure 5:**
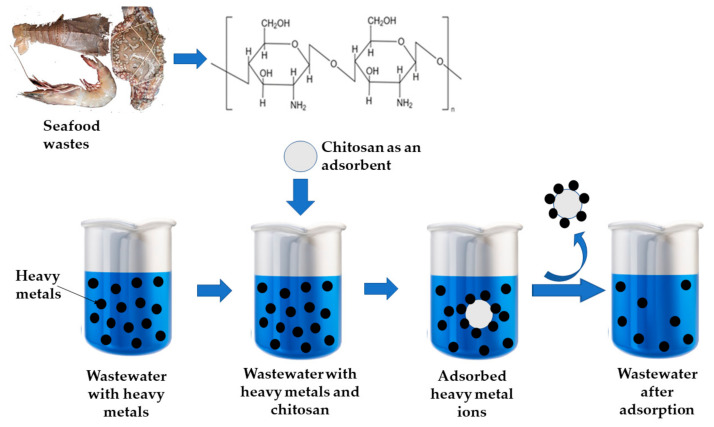
Chitosan is employed for removing heavy metals from wastewater.

Water treatment for heavy metal (As, Pb, Hg, Cd, Cu, Cr) and radionuclide (U, Se, Tc) removal has been accomplished using water-stable adsorbents made from metal-organic frameworks (MOF) and MOF-based composites. These materials possess remarkable porosity and surface area, facilitating adsorption and/or photocatalytic redox processes [83]. Figure 5 depicts CS’s ability to remove heavy metals from wastewater.

### 6.2. Chitosan Based Nanocomposites to Eliminate Heavy Metal Ions

The outstanding properties of nanomaterials, including high surface areas, numerous active sites, and exceptional sorption capabilities, make them highly valuable for treating wastewater [84,85,86]. In recent years, numerous studies have focused on producing magnetic CS-based nanocomposites for extracting metal ions [87,88,89]. Nano-absorbents made from magnetic CS nanoparticles or nanocomposites have been investigated for their effectiveness in removing metal ions from wastewater. The CS polymer chain contains an increased number of hydroxyl and amino groups, which make it highly selective and capable of strongly chelating metals, enabling efficient sorbent regeneration. As a result, CS nanoparticles have the potential to serve as a reusable absorbent for wastewater treatment [90].

Chitosan-citrate gel beads (CCGBs) containing N, O-carboxymethyl chitosan-coated magnetic nanoparticles (NOCC-MNPs) were developed, exhibiting outstanding adsorption capacity for Cu(II) ions (294.11 mg/g). The chelating ability of NOCC, resulting from the presence of hydroxyl, carboxyl, and amino groups in the carboxymethyl chitosan, was responsible for this result [91].

To enhance the mechanical properties of composite materials, a green and efficient approach involves adding different reinforcing fillers to the polymer matrix [92]. The SiO_2_/CS composite is capable of adsorbing heavy metal ions in solution, particularly As(V) and Hg(II), with a high performance (198.6 and 204.1 mg/g, respectively). SiO_2_ possesses stable chemical properties and good mechanical strength and can effectively prevent the adsorbents from agglomerating. In addition, CS NPs coated on SiO_2_ provide an abundance of functional groups (amino and hydroxyl groups) for heavy metal ions [93]. Incorporating nanofillers such as TiO_2_ can enhance the adsorption capacity of CS by modifying the polymer’s molecular network and increasing the surface area available for metal adsorption [94]. Razzaz et al. [94] used both entrapped and coating methods to produce chitosan/TiO_2_ nanofibers, which exhibited high adsorption capacities for Pb(II) and Cu(II) (ranging from 475.50 to 715.70 mg/g).

### 6.3. Mechanism

Based on the solute affinity, adsorption can occur in three types: physical (physisorption), ion exchange and chemical adsorption (chemisorption). Physical adsorption is the result of mainly Van der Waals attraction due to the electrostatic forces between the adsorbate and the surface of the adsorbent. Ion exchange adsorption depends on electrostatic forces occurring between ions retained on the surface, while chemisorption is the result of covalent bonds occurring via chemical interaction between adsorbate and adsorbent. Chemisorption is highly selective, i.e., it occurs between specific adsorptive and adsorbent species, particularly when the active sites are not blocked by previously adsorbed molecules [79].

CS and its nanocomposites can remove heavy metal ions through various mechanisms, such as electrostatic interaction, ionic exchange, metal chelation, and ion-pair formation [95,96]. The adsorption of CS/Fe_3_O_4_ NPs is mainly due to the ion exchange on the hydroxyl groups of CS/Fe_3_O_4_ NPs and the chelation action of the abundant amine groups in CS [90]. CS is a natural bio-sorbent for toxic metallic ions due to a large number of hydroxyl and amino groups that serve as sorption sites and the flexibility of the polymer chain structure that enables complexation with metal ions [97]. The chelation capacity of CS depends on the degree of deacetylation, distribution of –NH_2_ groups, the physical state of CS, the nature of the metal ion, and the pH of the solution [97,98]. Higher degrees of deacetylation result in better chelation [98].

CS is protonated in acidic media and exhibits electrostatic characteristics, and the adsorption occurs via an anion exchange mechanism [99]. To increase the density of sorption sites for metal ion adsorption, CS has been modified through the addition of new functional groups to the CS backbone, resulting in numerous CS derivatives [97].

### 6.4. Isothermal and Kinetic Model

The equilibrium data of metal ions sorption onto the synthesized CS-based adsorbents can be described using various models, including Freundlich, Langmuir, Redlich–Peterson, Dubinin–Radushkevich (D–R), and Sips isotherm [77,94]. These isotherm models are used to investigate the dominant adsorption mechanism and to calculate adsorption parameters [77].

The isotherm equations are given as follows;

(2) $$\mathrm{Langmuir}:q_{e}=\frac{q_{m}K_{L}C_{e}}{1+K_{L}C_{e}}$$

(3) $$\mathrm{Freundlich}:q_{e}=K_{F}C_{e}^{1/n}$$

(4) $$\mathrm{Sips}:q_{e}=\frac{q_{m}K_{S}C_{e}^{1/n}}{1+K_{S}C_{e}^{1/n}}$$

(5) $$\mathrm{Redlich}–\mathrm{Peterson}:q_{e}=\frac{PC_{e}}{1+\alpha C_{e}^{\beta }}$$

where, *q_e_*(mg/g) and *C_e_* (mg/L) are the equilibrium adsorption capacity and the equilibrium concentration of each ion in the solution, *q_m_* (mg/g) is the maximum adsorption capacity, *K_L_* (L/mg) is Langmuir equilibrium constant. *K_F_* [(mg/g)(L/mg)^1/n^] and *n* are the Freundlich equilibrium constant, which measures the heterogeneity of surface adsorption sites. The *n* value ranges from 1–10, indicating that adsorption is favorable. In the Sips isotherm equation, all these parameters keep their meaning. *P* (L/mg) and *α* (L/mg) are the isotherm constant of the Redlich–Peterson model, and *β* is the exponential value ranges from 0–1.

**Figure 6:**
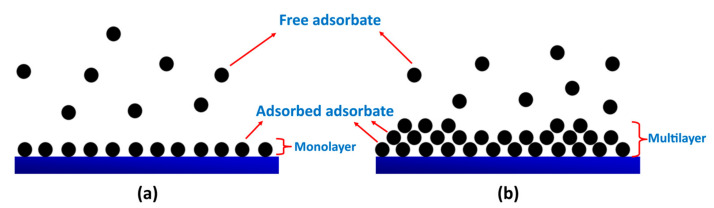
Schematic representation of monolayer and multilayer adsorption of heavy metals. (**a**) monolayer adsorption, (**b**) multilayer adsorption.

Langmuir and Freundlich are the most commonly adopted model to check the best isotherm fit as they are simple models as compared to the others [100]. Langmuir isotherm model assumes the monolayer surface adsorption on homogenous active sites; each active site can adsorb only a single molecule and reach the maximum adsorption capacity when all sites are occupied [77,91]. The Freundlich isotherm model indicates heterogeneous surface adsorption; both monolayer and multilayer adsorption occurs (Figure 6) [77,94].

Furthermore, adsorption kinetic indicates the rate of adsorption and determines the adsorption reaction mechanism. The pseudo-first-order and the pseudo-second-order kinetic models illustrate the rate of adsorption and its nature, such as chemical or physical adsorption, while intraparticle diffusion. The pseudo-first-order kinetic model suggests that the heavy metals are binding via physical adsorption, whereas the pseudo-second-order kinetic model indicates that the binding is achieved by chemical adsorption. Another model, intraparticle diffusion, gives an idea of whether the mass transfer (diffusion) within the pores limits the adsorption rate or not [55,77,100].

Mi et al. [91] reported that the adsorption of the Cu(II) by magnetic CS-citrate gel beads fitted well with the Freundlich model (R^2^ = 0.964), which suggests that adsorption occurs on irregular surfaces. Moreover, the adsorption kinetics followed the pseudo-second-order kinetic model, suggesting that Cu(II) is mainly adsorbed by chemisorption [91].

Magnetic graphene oxide/CS [75], Fe_3_O_4_/CS/polyethylenimine [101], Fe_3_O_4_/CS NPs [90], CS/vanillin CS/ortho-vanillin [97], CS/bentonite [102], and TiO_2_/CS [94] were well fitted with Langmuir isotherm providing a suggestion of monolayer adsorption of various heavy metals.

CS/EDTA complex was also well fitted to the monolayer Langmuir isotherm, with maximum adsorption capacities of 227.27–370.37 mg/g for Pb(II), Cd(II), and Cu(II). The adsorption followed the pseudo-second-order kinetics and was attributed to the electrostatic interactions between the metals and different functional groups (single bond –OH, –NH_2_, and –COOH) of the adsorbent and complexation with EDTA [103]. Fe_3_O_4_/CS NPs have a strong metal chelating capability, which may be attributed to the strong metal chelating capacity of CS, and the magnetic CS can be efficiently separated the external magnetic field from the media [73,90].

As shown in Table 4, most of the CS-based adsorbents obeyed the pseudo-second-order kinetic model, indicating that the adsorption mechanism of heavy metal is chemisorption, which may be achieved through chelation or electrostatic attraction.

### 6.5. Reusability or Regeneration

Regeneration and reusability are performed by repeating the adsorption and desorption cycles and are vital criteria in commercial applications, and provide economic and environmental benefits [75,99]. For regeneration or reuse of adsorbents, it can be obtained by washing with various desorption reagents, such as acids (HCl, H_2_SO_4_, HNO_3_, H_3_PO_4_), alkalis (NaOH, NH_4_OH), salts (NaCl, Na_2_CO_3_, Na_2_SO_4_, KNO_3_), and chelating (EDTA, Na_2_EDTA) agents followed by deionized water, and oven drying (60 °C for 1 h). [93,98]. Algethami et al. [104] reported that HCl (91.3%) was the best eluent for the desorption of Cd(II) compared to that of CH_3_COOH (47.48%) and HNO3 (87.26%).

The removal efficiency of Fe_3_O_4_/CS/PEI nanosorbent using NaOH as eluent was reported as above 90.0% after the 5th cycle [101]. The removal efficiency for heavy metals was reported as >90% even after the 5th cycle in Fe_3_O_4_/CS NPs [90,101], Magnetic thiolated/quaternized-CS [77], and CS-EDTA [103].

In contrast, a severe collapse also was (44%) observed in removal efficiency with CS/Fe_3_O_4_ NPs at the end of the fifth run due to a decline of the NPs mass and lower efficiency of CS modification after several times of washing as well as the irreversible adsorption on the adsorbent active sites or partial regeneration of Cr(IV) by NaOH solution [55]. In another study, compared to TiO_2_-entrapped CS nanofibers, more than 40% loss of total adsorption was reported after the 5th cycle in TiO_2_-coated CS nanofibers due to the physical loss of TiO_2_ NPs by acid cleavage [94]. Sutirman et al. [105] developed crosslinked CS grafted with methyl methacrylate (M-CS) for the removal of Cu(II) ions. The removal efficiency was significantly reduced to 80% after four consecutive adsorption-desorption cycles may be attributed to damage to the structure and the loss of active sites during the reusability processes using HNO_3_. Shahraki et al. [106] also reported adsorption capacity after 5 cycles as 118 mg/g, which was 51% of the initial value.

Table 4 summarizes the previous studies on CS composites for removing several heavy metals from aqueous solutions.

**Table 4:** Literature survey on CS composites for the removal of heavy metals from aqueous solutions.

Adsorbent	Adsorbate	Kinetics	Isotherm	pH	Temperature (°C)	Adsorption Capacity (mg/g)	Reference
CS/Fe_3_O_4_ NPs	Cr(VI)	PSO	L	3.37	298 K	162	[55]
Zeolitic imidazolate framework-67 (ZIF-67)/modified bacterial cellulose/CS aerogel	Cu(II)	PSO	-	6	25	200.6	[61]
	Cr(VI)					152.1	
Magnetic graphene oxide/CS (Fe_3_O_4_/GO/CS)	Ni(II)	PFO	L	6	25	80.48	[75]
Magnetic thiolated/quaternized-CS	Pb(II)	PSO	S	7	30	235.63	[77]
	As(III)					67.69	
	As(V)					66.27	
	Hg(II)					28.00	
	Cu(II)					33.99	
Fe_3_O_4_/CS NPs	Pb(II)	-	L	6	-	79.24	[90]
	Cd(II)					36.42	
N,O-carboxymethyl chitosan-coated magnetic nanoparticles (NOCC-MNPs)/chitosan-citrate gel beads (CCGBs)	Cu(II)	PSO	F			294.11	[91]
SiO_2_@CS	As(V)	PSO	F	6–7	298 K	198.6	[93]
	Hg(II)		L			204.1	
TiO_2_/CS	Cu(II)	PFO	L	6	45	526.5–715.7	[94]
	Pb(II)					475.5–579.1	
CS/vanillin CS/ortho-vanillin	Co(II)	PSO	L	4	30	5.899–7.651	[97]
Fe_3_O_4_/CS/polyethylenimine (PEI)	As(III)	PSO	L	6.7	30	77.61	[101]
	As(V)					86.50	
Polymer composite (CS-EDTA)	Pb(II)	PSO	L			370.37	[103]
	Cd(II)					243.90	
	Cu(II)					227.27	
Dimercaptosuccinic acid-functionalized magnetic CS (Fe_3_O_4_@CS@DMSA)	Cd(II)	PFO	L	7.6		314.12	[104]
Crosslinked CS grafted with methyl methacrylate (M-CS)	Cu(II)	PSO	L	4	RT	192.31	[105]
Magnetically modified CS/3,3-diphenylpropylimine methyl benzaldehyde (PPIMB)	Pb(II)	PSO	L			230.48	[106]
Magnetic xanthate-modified CS/polyacrylic acid	Cu(II)	PSO	L	5.5	30	206	[107]
	Cd(II)		-			178	
	Pb(II)		-			168	
	Co(II)		-			140	
Glucan/CS	Cu(II)	PSO	L	7	25	342	[108]
	Co(II)					232	
	Ni(II)					184	
	Pb(II)					395	
	Cd(II)					269	
CS/calcium alginate/bentonite	Pb(II)	PSO	R	5		434.89	[109]
	Cu(II)					115.30	
	Cd(II)					102.38	
CS microspheres/sodium alginate hybrid beads	Pb(II)	PFO	L			180	[110]
	Cr(VI)	PSO	L			16	
CS modified with carboxyl groups	Cu(II)		L	3.5	25	220.5	[111]
	Zn(II)					124.3	
AgNPs/GO/CS nanocomposite	Mn(II)	PSO	F	6	30	1605	[112]
Microfluidically-generated CS microspheres	Cu(II)	PSO	L	5.5	35	75.52	[113]
CS grafted UiO-66-NH2	Cu(II)	-	-	-	-	364.96	[114]
	Pb(II)					555.56	
CS-g-acrylamide-orange peel	Cr(VI)	PSO	F	4	28	178.34	[115]
	Cu(II)			5	28	181.88	

PFO, Pseudo-first-order; PSO, Pseudo-second-order; RT, Room temperature; F, Freundlich; L, Langmuir; R, Redlich–Peterson; S, Sips isotherm model.

## 7. Conclusions and Future Prospective

Heavy metals are a significant water pollutant and a serious threat to human health and the environment because of their non-biodegradability, carcinogenicity, and toxicity, even at low concentrations. Heavy metal pollution in water can result in severe health problems caused by the accumulation of toxic heavy metals through the food chain. Hence, it is necessary to remove heavy metals from the water before discharging it into the environment, ensuring safe water consumption and human activities.

CS-based materials have been extensively studied as eco-friendly and biodegradable adsorbents for water treatment. CS contains hydroxyl and amine groups in the glucosamine unit that facilitate metal adsorption through various mechanisms, such as metal chelation, electrostatic interaction, ionic exchange, and ion-pair formation. CS is soluble in acidic pH and insoluble in neutral and basic pH. The modification of CS through physical, chemical, and biological methods improves its physicochemical properties and metal adsorption capacity, making it a promising material for heavy metal removal from water.

CS-based materials have shown great potential as a sustainable solution for the removal of heavy metals from water. In the future, further research and development are needed to optimize the performance of these materials, such as improving their mechanical properties and enhancing their selectivity for specific metals. There is also a need to explore novel approaches for the synthesis and functionalization of chitosan-based materials, such as using green chemistry and biotechnology methods. Additionally, the scalability and cost-effectiveness of chitosan-based materials need to be addressed for their commercialization and widespread application. Overall, the future prospects of chitosan-based materials for heavy metal removal from water are promising, and continued research and development in this area could lead to a more sustainable and effective solution for water treatment.

## Figures and Tables

**Table 1:** Proximate composition of different chitin sources [45].

Sources	Protein (%)	Ash (%)	Chitin (%)	Moisture (%)	Lipid (%)
Shrimp shells	32.77	32.46	36.43	45.65	-
Shrimp shells *(P. longirostris)*	29.23	25.06	26.98	3.25	15.48
Shrimp shells (*Penaeus durarum*)	34.02	42.26	23.72	-	-
Insect cuticles (Cicada sloughs)	39.8	11.7	36.6	8.7	2.7
Crabs’ shells	16.68	66.58	16.73	-	-
Mussel shells	9.99	23.25	23.25	-	-
Squid gladius (*L. vulgaris*)	36.52	2.57	31.2	-	0..32

Adapted from [45].

**Table 2:** Deacetylation conditions in CS production.

Source	Demineralization	Deproteinization	Decolorization	Deacetylation	Degree of Acetylation (DA) or Degree of Deacetylation (DD) (%)	Reference
Shrimp shells	2.5 M HCl (1:10 solid: solvent ratio *w*/*v*) for 4 min under MW at 650 W power	NaOH (20%) under MW irradiation at 500 W for 8 min.	-	30% NaOH for 12 min at 500 W	23.4% DA	[46]
Shrimp shells	HCl (7%) at ambient temperature for 24 h.	NaOH (10%) at ambient temperature for 24 h.	Ethanol for 6 h	NaOH (50% *w*/*v*) at a boiling temperature in the N_2_ atmosphere, Repeated twice	78% DD	[55]
Shrimp shells	1.5% HCl (1:30 *w*/*v*) for 20 h at room temperature	NaOH (5%) at 90 °C for 24 h (solvent: shell ratio 12:1, *v*/*w*).	Acetone (99.5%) at room temperature for 24 h.	50% NaOH (15%, *w*/*v*) at 60 °C for 8 h.	-	[56]
Giant freshwater prawn carapace	1 M HCl (1:10 solid: liquid ratio) at 60 °C, 250 rpm for 2 h.	1 M NaOH (1:10 solid: liquid ratio) at 100 °C, 250 rpm for 2 h.	95% ethanol (1:5 mass: volume ratio) for 30 min at ambient temperature	60% NaOH (1:10 solid: liquid ratio), at 120 °C, 250 rpm for 2 h.	85.2%	[57]
Prawn shells	1 M HCl (1:16 solid: liquor ratio) at 100 °C for 4 h.	1 M NaOH (1:16 solid: liquid ratio) at 100 °C for 4 h.		50% NaOH (1:30 solid: liquid ratio), in presence of ethanol, at 80 °C for 4 h.	89% DD	[58]
Crab shell	2.5% (*w*/*v*) HCl at 1:20 (*w*/*v*, shell: solution), 20 °C for 6 h.	2% KOH at 1:20 (*w*/*v*, shell: solution), 90 °C for 2 h	Acetone for 10 min	40% NaOH at 1:15 (*w*/*v*, chitin: solution) at 105 °C for 2 h.	53.42% DD	[59]

**Table 3:** Frequently used solvents for chitin and CS.

Chitin/CS	Solvents	References
Chitin	Dimethylformamide + lithium chloride, Diethylformamide + lithium chloride, Hexafluoroisopropanol, Hexafluoroacetone + sequihydrate, 1,2-Chloroethanol + sulphuric acid, high concentrated organic acids (HCl, H_2_SO_4_, H_3_PO_4_)	[65,67,68]
CS	Aqueous citric acids, acetic acid, lactic acid, formic acid, glutamic acid, HCl acid	

## Data Availability

Not applicable.
